# Verstetigung von Implementierungsprozessen in der stationären Langzeitpflege: Nachhaltigkeit des Expertenstandards *Beziehungsgestaltung in der Pflege von Menschen mit Demenz*

**DOI:** 10.1007/s00391-023-02266-0

**Published:** 2023-12-21

**Authors:** Carolin Mirbeth

**Affiliations:** Fakultät für Soziale Arbeit, Bereich Pflegewissenschaft, Katholische Universität Eichstätt-Ingolstadt, Kapuzinergasse 2, 85072 Eichstätt, Deutschland

**Keywords:** Qualität der Gesundheitsversorgung, Expertenstandard, Implementierungswissenschaft, Programmevaluation, Evidenzbasierte Pflege, Quality of health care, Practice guideline, Implementation science, Program evaluation, Evidence-based nursing

## Abstract

**Hintergrund:**

Pflegeeinrichtungen stehen aufgrund von Evidenzbasierungsforderungen vor großen Herausforderungen. Sie sind aufgefordert, neue Interventionen wie Expertenstandards einzuführen und Alltagsroutinen an neue Erkenntnisse anzupassen. Wenn aber fortlaufend neue Interventionen in den Einrichtungen implementiert werden, ist deren Nachhaltigkeit fraglich.

**Ziel der Arbeit:**

Ziel war es, zu eruieren, wie nachhaltig der Expertenstandard *Beziehungsgestaltung in der Pflege von Menschen mit Demenz* in Einrichtungen der stationären Langzeitpflege implementiert ist.

**Material und Methoden:**

Es wurden qualitative, leitfadengestützte Interviews mit Personen aus der stationären Langzeitpflege, orientiert am Vorgehen des problemzentrierten Interviews, geführt. Die Befragten waren an der modellhaften Implementierung des Expertenstandards *Beziehungsgestaltung in der Pflege von Menschen mit Demenz* beteiligt. Die Daten wurden anschließend mithilfe der qualitativen Inhaltsanalyse ausgewertet.

**Ergebnisse:**

Die Befragten schätzen die nachhaltige Umsetzung unterschiedlich ein. Einige bewerten die Nachhaltigkeit des Expertenstandards in ihrer Einrichtung als gelungen, andere wiederum sehen Verbesserungspotenzial. Nach Auffassung der Teilnehmenden wird die Verstetigung von Implementierungsprozessen durch verschiedene Faktoren beeinflusst. Diese betreffen die Einrichtung, den externen Kontext, die Intervention, den Implementierungsprozess und die Personen, die an der Implementierung beteiligt sind.

**Diskussion:**

Auf Basis der identifizierten Faktoren lassen sich zentrale Bedarfe für die Pflegepraxis, für die Wissenschaft und Forschung sowie Politik und Gesetzgebung ableiten, damit die Nachhaltigkeit von Expertenstandards und weiteren evidenzbasierten Interventionen gewährleistet und ggf. optimiert wird.

## Abstract (en)

**Background:**

Nursing institutions are facing many challenges due to evidence-based requirements. For example, they are required to introduce new interventions such as expert standards and thus adapt routine practices to new findings; however, if new interventions are continuously implemented in the facilities their sustainability is questionable.

**Objective:**

The aim was to find out how sustainably the expert standard “Relationship management in the care of people with dementia” is implemented in residential long-term care institutions.

**Material and methods:**

Qualitative, guideline-based interviews were conducted with persons from residential long-term care, oriented to the procedure of the problem-centered interview. The interviewees were involved in the model implementation of the expert standard “Relationship management in the care of people with dementia”’. The data were then analyzed using qualitative content analysis.

**Results:**

The participants assessed the sustainable implementation in different ways. Some rated the sustainability of the expert standard in their institution as successful, while others see potential for improvement. According to the participants, the sustainability of continuation of implementation processes is influenced by various factors. These relate to the institution, the external context, the intervention, the implementation process, and the people involved in the implementation.

**Conclusion:**

On the basis of the factors identified, key needs can be derived for nursing practice, for science and research as well as for politics and legislation, so that the sustainability of expert standards and other evidence-based interventions can be ensured and, if necessary, optimized.

## Hintergrund

Die Forderung nach einer Evidenzbasierung der Pflegepraxis [1] geht mit einer stetig steigenden Zahl von Forschungsprojekten einher und stellt die Pflegepraxis zunehmend vor Herausforderungen [8, 17, 18]. Diese beschäftigt sich bereits fortlaufend mit der Implementierung theoriebasierter Konzepte. Die Praxisakteur*innen müssen sich mit Implementierungsprozessen befassen und diese bestmöglich gestalten, damit die damit verbundenen Änderungen in den Alltag der Einrichtung integriert werden können [17]. Wenn die Pflegepraxis aber ständig mit der Implementierung von Veränderungsprozessen beschäftigt ist, stellt sich die Frage, wie nachhaltig die einzelnen Konzepte, Standards und wissenschaftlichen Befunde eingeführt und umgesetzt werden. Es ist nicht nur wichtig, dass die Neuerungen in die Praxis gelangen, sondern auch, dass sich diese nachhaltig, d. h. dauerhaft, in den Einrichtungen etablieren [2]. Es sollte nicht nur die Einführung evidenzbasierter Interventionen forschungsrelevant sein, sondern auch deren Verstetigung. Kritisch zu sehen ist allerdings, dass die Nachhaltigkeit von Implementierungsprozessen nur selten im Mittelpunkt der Forschung steht [11, 15, 20]. Dies mag auch die Expertenstandards des Deutschen Netzwerks für Qualitätsentwicklung in der Pflege (DNQP) betreffen. Unter dem Aspekt, dass regelmäßig neue Expertenstandards herausgegeben und in der Pflegepraxis implementiert werden, könnte dies mit einer Überforderung der Praxisakteur*innen einhergehen und sich negativ auf die Anwendung evidenzbasierter Interventionen und ihre Akzeptanz auswirken. Da aber Expertenstandards einen Beitrag zur Qualitätsentwicklung leisten [2], ist ihre dauerhafte Etablierung in den Einrichtungen essenziell.

## Zielsetzung und Fragestellung

Um die Nachhaltigkeit von Expertenstandards in den Blick zu nehmen, wurde der Expertenstandard *Beziehungsgestaltung in der Pflege von Menschen mit Demenz* ausgewählt. Dieser ist seit rund 4 Jahren für die Pflegepraxis zugänglich [2] und spricht ein Thema an, das aufgrund der steigenden Zahl von Menschen mit Demenz zunehmend an Relevanz gewinnt [4]. Die im Folgenden beschriebene Untersuchung beschäftigt sich mit der Frage, wie nachhaltig der Expertenstandard *Beziehungsgestaltung in der Pflege von Menschen mit Demenz* in der stationären Langzeitpflege implementiert ist. Die Studie soll Hinweise darauf liefern, inwiefern die stationären Langzeitpflegeeinrichtungen den thematisierten Expertenstandard zum Befragungszeitpunkt nutzen, und ob sich dieser dauerhaft in der Einrichtung etablieren konnte. Der vorliegenden Studie wird die Nachhaltigkeitsdefinition von Hoben und Bär [10] zugrunde gelegt. Diese definieren Nachhaltigkeit als „den Grad, in dem eine Intervention über die Projektphase hinaus über einen längeren Zeitraum aufrechterhalten wird“ [10, S. 229].

## Methodisches Vorgehen

Die Forschungsfrage wurde mithilfe einer qualitativen Querschnittstudie beantwortet und in Form von problemzentrierten Interviews [21] umgesetzt, um die Erfahrungen und die Sichtweise der Personen, deren Einrichtung an der modellhaften Implementierung des thematisierten Expertenstandards beteiligt war, zu ermitteln und deren Blickwinkel auf die Thematik der Nachhaltigkeit von Implementierungsprozessen am Beispiel des Expertenstandards herauszustellen. Um den aktuellen Forschungsstand zu eruieren, ging der Datenerhebung eine Literaturrecherche in den (Fach)Datenbanken MEDLINE (via PubMed), CINAHL und Cochrane Library voraus.

### Feldzugang und Sample

In das Sample eingeschlossen wurden Einrichtungen, die an der modellhaften Implementierung des Expertenstandards beteiligt waren. Insbesondere sollten die Befragten aktiv an ebendieser teilgenommen haben, wie es beispielsweise bei den projektverantwortlichen Personen resp. Projektgruppenmitgliedern der Fall ist. Die Teilnehmenden wurden über die Veröffentlichung des DNQP zur modellhaften Implementierung des entsprechenden Expertenstandards [5] rekrutiert. Dieser konnten 11 Einrichtungen der stationären Langzeitpflege mit der jeweiligen projektverantwortlichen Person sowie der zugehörigen Führungsperson entnommen werden.

Insgesamt wurden 15 Personen aus 8 Einrichtungen in die Befragung eingeschlossen. Bei 5 Teilnehmenden handelt es sich um die projektverantwortliche Person; 9 Teilnehmende waren als Projektgruppenmitglieder an der Implementierung beteiligt. Lediglich eine Person kam erst nach der modellhaften Implementierung als Qualitätsbeauftragte in die Einrichtung. Die Befragten sind alle weiblich und haben unterschiedliche Funktionen inne. Vier Teilnehmerinnen sind als Einrichtungsleitung und weitere 3 als Pflegedienstleitung in den Einrichtungen tätig. Neben einer Leitung des Sozialen Dienstes beteiligten sich auch eine stellvertretende Pflegedienstleitung und eine leitende Referentin eines trägerinternen Ressorts an den Interviews. Des Weiteren setzte sich die Stichprobe aus einer Qualitätsbeauftragten, einer Diplom-Gerontologin, einer Projektmanagerin sowie einer Pflegefachkraft ohne und einer mit gerontopsychiatrischer Fachweiterbildung zusammen.

### Datenerhebung und -analyse

Die Datenerhebung erfolgte mithilfe eines leitfadengestützten problemzentrierten Interviews [21]. Der Leitfaden gliederte sich zunächst in 2 Teilbereiche: die Erfahrung der Teilnehmenden während der modellhaften Implementierung des Expertenstandards und den aktuellen Stand der Verwendung des Expertenstandards in der Einrichtung. Zudem wurden die Einflussfaktoren der Nachhaltigkeit, die mithilfe einer Literaturrecherche identifiziert werden konnten, sowie implementierungsbezogene Outcomes [16] als Themenbereiche gewählt. Insgesamt konnten zwischen Juli und August 2022 zehn Interviews geführt werden. Davon wurden in 5 Interviews 2 Teilnehmer*innen gemeinsam befragt. Die restlichen Interviews konnten im Einzelgespräch durchgeführt werden. Bei einer Einrichtung war es möglich, 3 Einzelgespräche zu führen. Die Interviews wurden entweder vor Ort in den Einrichtungen (1), virtuell über Videokommunikationstools (6) oder telefonisch (3) geführt und nach Zustimmung der Teilnehmenden mithilfe eines Audioaufnahmegeräts aufgezeichnet.

**Figure 1:**
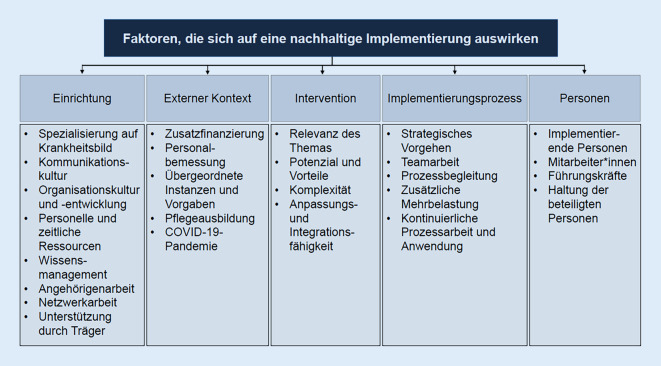

Die Transkription erfolgte in Anlehnung an Dresing und Pehl [6]. Die Daten wurden anhand der qualitativen Inhaltsanalyse nach Mayring [14], speziell der zusammenfassenden Inhaltsanalyse mit induktiver Kategorienbildung, ausgewertet. Die Datenauswertung erfolgte computerunterstützt mithilfe der Datenanalysesoftware MAXQDA (Version 2022). In 3 Durchgängen wurde das Datenmaterial so weit reduziert, bis ein Kategoriensystem aus 5 zentralen Oberkategorien und 26 Unterkategorien gebildet werden konnten (Abb. 1).

### Gütekriterien

Der Studie wurden die Gütekriterien qualitativer Forschung nach Mayring [13] zugrunde gelegt. Die Gütekriterien „Verfahrensdokumentation“, „argumentative Interpretationsabsicherung“, „Nähe zum Gegenstand“ und „kommunikative Validierung“ wurden erfüllt. Nicht umgesetzt werden konnte das Gütekriterium „Triangulation“, da sich lediglich auf eine Datenerhebungsmethode konzentriert wurde.

## Ergebnisse

Die Teilnehmenden gaben im Laufe des Interviews eine Einschätzung darüber ab, ob der Expertenstandard zum Befragungszeitpunkt nachhaltig in der Einrichtung etabliert ist. Einige beschrieben, dass der Expertenstandard in ihrer Einrichtung nachhaltig umgesetzt wurde, andere wiederum sahen Verbesserungspotenzial. Durch die Interviews konnten entscheidende Momente der Nachhaltigkeit identifiziert werden; diese werden im Folgenden zusammenfassend dargestellt. Eine umfassende Darstellung aller Einflussfaktoren findet sich in Abb. 1.

### Einrichtungsbezogene Faktoren

Vorteilhaft ist es, wenn Einrichtungen bereits auf das zugrunde liegende Krankheitsbild spezialisiert sind und in diesem Zusammenhang auf bestehende Einrichtungskonzepte und Vorwissen zum Thema des Expertenstandards zurückgreifen können. Zudem trägt eine offene Kommunikationskultur innerhalb der Einrichtung dazu bei, dass sich der Expertenstandard in der Einrichtung weiterentwickelt und in bestehende Strukturen einfließt. Hierzu sind Austauschmöglichkeiten für Mitarbeitende, wie z. B. Fallbesprechungen, entscheidend. Förderlich ist weiter eine flexible Organisationskultur, die sich den Expertenstandardinhalten anpassen lässt und nicht durch starre Strukturen und Abläufe begrenzt ist. Neben ausreichend personellen und zeitlichen Ressourcen ist auch ein ausgereiftes Wissensmanagement entscheidend für eine nachhaltige Umsetzung. Hierbei sollten Schulungen für alle Personen in der Einrichtung kontinuierlich und dauerhaft angeboten sowie an den Bedürfnissen der Teilnehmenden ausgerichtet werden. Auch das Vorleben der Inhalte ist entscheidend. Zum Beispiel ist es essenziell, dass das Wissen und die Inhalte des Expertenstandards bei vorhanden Sprachbarrieren durch erfahrene Kolleg*innen vorgelebt werden. Um den Expertenstandard nach der erfolgreichen Implementierung aufrechtzuerhalten, ist die Einarbeitung neuer Mitarbeiter*innen ein wichtiger Punkt im Wissensmanagement der Einrichtung. Kennen diese den Expertenstandard nicht, werden sie diesbezüglich geschult. Neue Mitarbeiter*innen müssen mitgenommen und die Inhalte des Expertenstandards vorgelebt werden. Neben dem Einbezug der Angehörigen in alle Prozesse, ist es weiter vorteilhaft, mit anderen Einrichtungen in den Austausch zu treten und eine gute Netzwerkarbeit zu pflegen.

### Externe Kontextfaktoren

Einen Einfluss auf die Nachhaltigkeit haben externe Kontextfaktoren, welche die Einrichtungen von außen, beispielsweise durch Politik oder Gesetzgebung, beeinflussen. Förderlich ist es, wenn Einrichtungen mithilfe einer Zusatzfinanzierung, z. B. durch Rahmenverträge oder Förderprogramme, zusätzliche Arbeitskräfte außerhalb des Stellenplans einsetzen können. Hemmend ist, wenn zu wenig Personal in den Einrichtungen eingesetzt werden kann. Dies resultiert aus unzureichenden Personalschlüsseln und ungenügender Finanzierung des Pflegepersonals. Ein besserer Personalschlüssel und eine optimierte Bezahlung würden die Situation insgesamt erleichtern und die Nachhaltigkeit von Interventionen unterstützen. Zudem erhöhen externe Vorgaben, wie beispielsweise Qualitätsprüfungsverfahren, den Arbeitsaufwand, der wiederum die ohnehin knappen Ressourcen begrenzt. Die nachhaltige Implementierung des Expertenstandards ist durch viele verschiedene Themen und Konzepte, die ebenso Veränderungsprozesse bedürfen, gefährdet.

### Interventionsbezogene Faktoren

Unter diese Oberkategorie fallen alle Aspekte, die in direktem Zusammenhang mit der zu implementierenden Intervention stehen. Zum einen ist es entscheidend, dass das Interventionsthema für Menschen in der Praxis von Relevanz ist. Zum anderen muss die Intervention klare Potenziale und Vorteile für alle Beteiligten mit sich bringen. Hemmend ist, wenn die Intervention zu komplex gestaltet und formuliert ist und von den Praxisakteur*innen nicht verstanden wird. Daneben haben Pflegekräfte häufig aufgrund der Komplexität von Expertenstandards Befürchtungen, dass der Expertenstandard zu theoretisch und zu abstrakt ist, als dass sie wirklich davon profitieren können. Außerdem ist es wichtig, dass sich die Intervention an die Gegebenheiten in der Praxis anpassen und in bestehende Strukturen integrieren lässt.

### Prozessbezogene Faktoren

Im Hinblick auf prozessbezogene Faktoren ist ein strategisches Vorgehen während der Implementierung entscheidend. Optimal ist es, den Expertenstandard schrittweise an die Spezifitäten der jeweiligen Einrichtung adaptiert zu implementieren und fortlaufend zu evaluieren. Weiter ist es förderlich, auf bestehende Strukturen zurückzugreifen und die Implementierung nicht allein, sondern im multiprofessionellen Team, z. B. durch eine Projektgruppe, durchzuführen. Wichtig ist, dass diese berufsgruppenübergreifend gestaltet wird, da alle Mitarbeitenden in der stationären Langzeitpflege auf unterschiedliche Weise für die Versorgung von Menschen mit Demenz zuständig sind und täglich mit ihnen in Kontakt treten. Die Regelmäßigkeit der Projektgruppentreffen ist ein weiterer wichtiger Faktor. Der Einbezug aller Mitarbeitenden in den gesamten Implementierungsprozess sowie eine interne bzw. externe Prozessbegleitung sind weitere förderliche Faktoren. Letztere erleichtert den Praxisakteur*innen die Umsetzung des Expertenstandards und leistet einen Beitrag zu seiner dauerhaften Etablierung. Im Sinne einer nachhaltigen Umsetzung sehen die Befragten die erneute Kontaktaufnahme – beispielsweise bei Unklarheiten oder Rückfragen – sowie eine kontinuierliche Begleitung des DNQP auch nach Abschluss der modellhaften Implementierungsphase als gewinnbringend an. Um eine dauerhafte Etablierung zu erreichen, müssen die Inhalte des Expertenstandards kontinuierlich angewandt und immer wieder in den Fokus gebracht werden.

### Personenbezogene Faktoren

Ein letzter wichtiger Faktor sind die beteiligten Personen in den Einrichtungen – d. h. die Mitarbeitenden, Führungskräfte und Projektgruppenmitglieder. Bei Letzteren ist es entscheidend, dass diese vom Team akzeptiert werden. Auch Mitarbeitende beeinflussen mit ihren Merkmalen und Einstellungen Veränderungsprozesse. Wichtig ist, dass sie sich auf Veränderungen einlassen, Weiterentwicklungsbereitschaft zeigen sowie den Expertenstandard akzeptieren und verstehen. Weiter ist es positiv, wenn Mitarbeitende eine wertschätzende Haltung untereinander einnehmen, sich gegenseitig unterstützen und voneinander lernen. Zudem sind ihre Zufriedenheit und ihr Motivationsgrad für eine nachhaltige Implementierung essenziell. Aber auch die Führungsqualität der Leitungskräfte nimmt Einfluss auf die Nachhaltigkeit. Förderliche Aspekte sind die Berücksichtigung der Mitarbeiter*innenbedürfnisse sowie die Begleitung und Unterstützung der Mitarbeiter*innen durch die Leitungskräfte. Letztere verwirklichen die Führungskräfte beispielsweise durch transparente Reflexion, durch ein regelmäßiges Gesprächsangebot und Feedback. Entscheidend ist, dass Leitungskräfte die Prozesse begleiten und die Mitarbeiter*innen in der Lösungsentwicklung unterstützen. So können sich Interventionen langfristig in der Praxis etablieren. Leitungskräfte müssen ihre Vorbildfunktion wahrnehmen und die Inhalte des Expertenstandards vorleben. Sie sind in alle Prozesse miteingebunden und dürfen sich nicht herausnehmen. Neben der Führungsqualität ist auch der Rückhalt der Führungsebene entscheidend. Die Befragten sehen es als wichtig an, dass die Leitungskräfte hinter der Umsetzung des Expertenstandards stehen. Ist dies nicht der Fall, wird sich der Expertenstandard in der Einrichtung nicht etablieren.

## Diskussion

Die erzielten Ergebnisse bieten einen Überblick über Faktoren, die sich auf die Nachhaltigkeit der Implementierung des Expertenstandards *Beziehungsgestaltung in der Pflege von Menschen mit Demenz* auswirken, und leisten einen Beitrag zu verstehen, welche Faktoren relevant sind, um den Expertenstandard nachhaltig in der stationären Langzeitpflege zu implementieren. Aufgrund der vorhandenen Forschungslücke zum Thema Nachhaltigkeit von Expertenstandards liefert die hier dargestellte Untersuchung erste Erkenntnisse zur Verstetigung von Expertenstandards in der stationären Langzeitpflege und hilft zu verstehen, welche Aspekte Beachtung finden sollen, damit der thematisierte Expertenstandard zur Routine in den Einrichtungen wird. Die befragten Personen beschreiben eine Vielzahl von Faktoren, die Veränderungsprozesse beeinflussen und die auch in der Literatur zum Thema Dauerhaftigkeit komplexer Interventionen [7, 9, 19, 20] sowie im Consolidated Framework for Implementation Research (CFIR, [3]) zu finden sind. Gemeinsamkeiten liegen beispielsweise auf der Ebene der externen Kontextfaktoren und auf der Ebene der Einrichtung [3]. Im CFIR werden als Einflussfaktoren einer erfolgreichen Implementierung auf Ebene der Einrichtung die Organisationskultur, die Netzwerkarbeit und eine niedrige Personalfluktuation sowie im Zusammenhang mit externen Kontextfaktoren der Einfluss von externen Regelungen und Vorgaben identifiziert. Ebenfalls wird im CFIR ausgeführt, dass die zu implementierende Intervention verschiedene Merkmale aufweisen muss, um erfolgreich umgesetzt zu werden [3]. Merkmale wie die Komplexität, Anpassungsfähigkeit und Relevanz finden sich sowohl im CFIR als auch in der vorliegenden Arbeit. Außerdem lassen die Ergebnisse Analogien zu den Aussagen der Extended Normalization Process Theory [12] erkennen. Demnach ist es entscheidend, dass Einrichtungen den beteiligten Akteur*innen Zugang zu Wissen ermöglichen und Informationen über die komplexe Intervention bereitstellen. Die kognitiven Ressourcen der Beteiligten sind entscheidend, damit der Übergang in die Routine gelingt. Die Einrichtung ist dafür zuständig, diese entsprechenden Ressourcen bereitzustellen [12]. Die Theorie bestätigt die vorliegenden Ergebnisse außerdem in dem Punkt, dass den beteiligten Akteur*innen im sozialen System eine zentrale Rolle zukommt. Damit sich eine komplexe Intervention nachhaltig umsetzen lässt und zur Routine werden kann, müssen die Beteiligten ein gewisses Potenzial aufweisen. Sie sollten über ausreichend Motivation verfügen, die komplexe Intervention im Alltag anzuwenden und umzusetzen [12]. Ein Faktor, den die Befragten als wesentlich für eine nachhaltige Implementierung sehen und der bisher nicht in der Literatur beschrieben ist, ist die Spezialisierung der Einrichtung auf das Krankheitsbild. Die Quintessenz ist, dass es förderlich ist, wenn Einrichtungen bereits vor der Implementierung mit der Implementierungsthematik vertraut sind und möglicherweise bereits ein einrichtungsbezogenes Konzept im Hinblick auf Menschen mit Demenz aufweisen. Ein weiteres Alleinstellungsmerkmal ist der Einbezug der Angehörigen in den Implementierungsprozess wie z. B. durch Informationsveranstaltungen und durch transparente Gestaltung der Veränderungsprozesse. Hilfreich sind außerdem das Angebot von Angehörigenberatungen, -begleitungen und -gesprächen sowie ein speziell für Angehörige angepasstes Schulungsangebot zum Thema Demenz. Dies scheint v. a. für die stationäre Langzeitpflege ein wichtiger Faktor zu sein, da die Angehörigen ein wesentlicher Bestandteil der gesamten Pflege- und Betreuungsorganisation sind. Darüber hinaus stellt die Prozessbegleitung einen zentralen Punkt dar. Alle teilnehmenden Einrichtungen profitierten von einer Begleitung durch das DNQP, externe Berater*innen oder hauseigene Stabsstellen. Die Forderung nach einer kontinuierlichen Begleitung der Projekteinrichtungen sowie einer Begleitung aller Pflegeeinrichtungen geht jedoch über die Aufgaben des DNQP bzw. der anderen Beteiligten hinaus. Da aber die Prozessbegleitung einen essenziellen Einflussfaktor der dauerhaften Etablierung des Expertenstandards darstellt, wäre es angebracht, bestehende externe Strukturen zu erweitern oder neue Strukturen zu schaffen. Ein Aspekt, der insbesondere in der Umsetzung des thematisierten Expertenstandards von Bedeutung zu sein scheint, ist die personenzentrierte Haltung der beteiligten Personen. Dies mag v. a. daran liegen, dass der Expertenstandard an sich ein Thema adressiert, das eine Haltungsentwicklung fordert und mit sich bringt. Es braucht also auch eine personenzentrierte Haltung bzw. einen Entwicklungsprozess ebendieser, um den Expertenstandard nachhaltig umzusetzen. Darüber hinaus zeigen die Ergebnisse, dass der Fokus mehr auf zentrale und wesentliche Fragen gelegt werden soll, bei denen dann aber die Nachhaltigkeit im Mittelpunkt steht. Insgesamt bedarf das Thema der Nachhaltigkeit von Expertenstandards weiterer empirischer Untersuchungen, um das Phänomen umfassend zu verstehen.

## Limitationen

Die Wahl des methodischen Vorgehens wird zur Beantwortung der Forschungsfrage als geeignet eingeschätzt. Alternativ wird ein quantitativer Ansatz als passend angesehen. Diese Vorgehensweise hätte vermutlich zu abweichenden Ergebnissen geführt. Aufgrund der Besonderheiten und settingspezifischen Unterschiede ist eine Übertragung der Ergebnisse auf andere Pflegesettings außerhalb der stationären Langzeitpflege nicht möglich. Zudem beziehen sich die Ergebnisse speziell auf den thematisierten Expertenstandard. Eine vollständige Übertragung auf andere Expertenstandardthemen ist auszuschließen. Allerdings ist anzunehmen, dass sich einige Aspekte auch im Zusammenhang mit anderen Expertenstandardthemen identifizieren lassen. Limitierend ist darüber hinaus der fehlende Einbezug der Bewohner:innen.

## Fazit für die Praxis

- Praxisakteur*innen können Einfluss auf die Nachhaltigkeit nehmen, indem sie den Implementierungsprozess strategisch gestalten und den Expertenstandard gemeinsam im multiprofessionellen Team implementieren.
- Die Einrichtungen selbst sollten gewisse Merkmale, wie eine offene Kommunikationskultur oder flexible, anpassbare Organisationsstrukturen mitbringen. Führungskräfte und Träger können darauf hinwirken, dass diese Merkmale in den Einrichtungen gegeben sind.
- Ausreichend personelle und zeitliche Ressourcen sowie ein kontinuierliches Schulungsangebot zum Thema des Expertenstandards sind wichtige Faktoren einer nachhaltigen Umsetzung. An diesen Stellen können die Träger der Einrichtungen ansetzen, um die dauerhafte Etablierung zu beeinflussen.
- Die Praxis profitiert von einer Praxisbegleitung zur Implementierung und Verstetigung.
