# Erratum zu: Verstetigung von Implementierungsprozessen in der stationären Langzeitpflege

**DOI:** 10.1007/s00391-024-02292-6

**Published:** 2024-02-29

**Authors:** Carolin Mirbeth

**Affiliations:** Fakultät für Soziale Arbeit, Bereich Pflegewissenschaft, Katholische Universität Eichstätt-Ingolstadt, Kapuzinergasse 2, 85072 Eichstätt, Deutschland

**Erratum zu:**

**Z Gerontol Geriat 2023**

10.1007/s00391-023-02266-0

Der Artikel „Verstetigung von Implementierungsprozessen in der stationären Langzeitpflege“ von Carolin Mirbeth wurde ursprünglich am 21. Dezember 2023 mit „Open Access“ online auf der Internetplattform des Verlags unter der Lizenz Creative Commons Attribution license 4.0 veröffentlicht.

 Nach der Veröffentlichung in Band 57 Heft 1 pp. 21–26 entschied sich die Autorin den „Open Access“ zu stornieren. Das Urheberrecht des Artikels wurde deshalb am 7. Februar 2024 in ©The Author(s), under exclusive licence to Springer Medizin Verlag GmbH, ein Teil von Springer Nature, 2024 unter Vorbehalt aller Rechte geändert.

